# Motor Neuronopathy With Widespread Fasciculations in MCM3AP‐Related Disorder: Clinical and Muscle MRI Insights

**DOI:** 10.1111/jns.70112

**Published:** 2026-03-12

**Authors:** Ana Flávia Andrade Lemos, Rodrigo Siqueira Soares Frezatti, Antônio Carlos dos Santos, Pedro José Tomaselli, Wilson Marques

**Affiliations:** ^1^ Department of Neurosciences and Behavior Sciences. School of Medicine of Ribeirão Preto University of São Paulo Ribeirão Preto Brazil; ^2^ Instituto Nacional de Ciência e Tecnologia Em Saúde Mental Digital. Chamada CNPq/SECTICS/CAPES/FAPs N° 46/2024 Porto Alegre Brazil

**Keywords:** case report, MCM3AP, motor neuronopathy, muscle MRI, non 5q SMA

## Abstract

**Background:**

Biallelic pathogenic variants in MCM3AP, encoding the germinal center–associated nuclear protein (GANP), have been linked to autosomal recessive peripheral neuropathies variably accompanied by cognitive impairment and multisystem involvement. To date, anterior horn cell involvement has not been documented in association with MCM3AP‐related disorders.

**Objective:**

To describe a patient with biallelic MCM3AP variants presenting with a motor neuronopathy phenotype and to provide the first whole‐body muscle MRI characterization associated with this gene.

**Methods and Results:**

A 53‐year‐old woman born to non‐consanguineous parents presented with early‐onset motor neuronopathy and lifelong learning difficulties. Neurological examination revealed generalized areflexia and widespread fasciculations without sensory abnormalities. Electroneuromyography demonstrated diffuse mixed acute‐on‐chronic denervation process. Whole‐body muscle MRI showed a selective non–length‐dependent pattern of fatty infiltration. Whole‐exome sequencing identified two likely pathogenic heterozygous variants in the MCM3AP gene.

**Standard Protocol Approvals, Registrations, and Patient Consents:**

According to the policies of our institution, single‐patient case reports do not require review or approval by the institutional ethics committee. Written informed consent for participation and for publication of clinical information, photographs, electrophysiological data, and muscle MRI images was obtained from the patient. No clinical trial registration was applicable.

**Conclusion:**

This case extends the phenotypic spectrum of MCM3AP‐related disorders to include a slowly progressive, non‐syndromic motor neuronopathy with electrophysiological evidence of active denervation and distinctive MRI findings. These observations highlight the hidden boundaries between hereditary motor neuropathies and anterior horn cell diseases, emphasizing the need for integrated clinical, neurophysiological, and genetic evaluation.

## 1 Introduction

Biallelic pathogenic variants in the MCM3AP gene have been linked to autosomal recessive peripheral neuropathies, often accompanied by variable degree of intellectual disability [1]. The clinical spectrum is heterogeneous, ranging from early‐onset progressive sensory loss with weakness and distal muscle atrophy to additional features such as seizures, scoliosis, obesity, primary ovarian failure, and, more rarely, episodic manifestations mimicking periodic paralysis [2, 3]. To date, no evidence of anterior horn cell involvement or findings consistent with pure motor neuron disorder have been described in association to MCM3AP‐related disorders.

The classification of hereditary motor neuropathies remains a major challenge, as clinical and electrophysiological features often overlap with anterior horn cell disorders. Distal hereditary motor neuropathies (dHMN) typically present as a length‐dependent slowly progressive axonopathy affecting distal motor neurons, with relative sparing of sensory fibers [4]. In contrast, genetic anterior horn cell diseases as spinal muscular atrophy (SMA) and amyotrophic lateral sclerosis (ALS) show non–length‐dependent weakness with variable proximal or diffuse distribution, sometimes accompanied by additional neurological or systemic features of differing severity [5].

Electromyography (EMG) typically shows chronic denervation and reinnervation in SMA, and active denervation associated to reinnervation in ALS; however, these distinctions often overlap [6, 7]. Genetic and phenotypic overlap between dHMN, SMA, and ALS underscores that clinical assumptions about pathophysiology may not always correspond to electrophysiological or molecular findings.

Clinically, pure SMA is characterized by exclusive lower motor neuron involvement, whereas ALS shows combined upper and lower motor neuron dysfunction.

Approximately 95% of SMA cases result from biallelic loss of function variants in SMN1 (5q13.2), while the remaining are attributed to pathogenic variants in genes outside 5q chromosome, hence the designation non‐5q SMA [5]. Importantly, this group may overlap clinically with ALS and dHMN, as in all three conditions lower motor neuron involvement is or may be the predominant manifestation (Harding 1993; [4]; Kiernan et al. 2021). Clinical features and electromyography alone are not always sufficient to determine the primary site of involvement in hereditary motor disorders as spontaneous activity and fasciculations, may be unexpectedly present in long‐standing conditions like in HSPB1 [8] and some forms of dHMN may show marked proximal weakness like in MORC2, PLEKHG5, VWA1 or SYT2 [9, 10].

Here, we sought to describe a patient harboring two heterozygous variants in MCM3AP, who presented with early onset non‐5q SMA and mild cognitive impairment, in whom diffuse spontaneous fasciculations were observed on EMG.

## 2 Case Presentation

A 53‐year‐old woman, born to healthy from non‐consanguineous parents, had normal neurodevelopment and achieved all expected milestones.

She presented in first decade of life with difficulty walking and frequent falls. During her second decade, she developed pes *cavus* and underwent multiple corrective foot surgeries. Over the following years, she experienced slow but progressive worsening. By the age of 15, hands weakness became evident, impairing fine motor skills. Since her third decade, she has shown reduced ambulation, weakness in neck extension and limited tongue protrusion, occasionally accompanied by choking episodes. Despite these limitations, she remains independent in daily activities and uses a wheelchair only for long‐distance mobility. She consistently had learning difficulties from early schooling, particularly in reading comprehension and mathematics.

Neurologic examination at age of 53 revealed a steppage gait with marked ankle dorsiflexion weakness and distal leg wasting. Sensory examination was normal, and plantar responses were flexor bilaterally.

She exhibited a distal‐predominant, symmetric, non–length‐dependent muscle weakness. Muscle strength, graded according to the Medical Research Council (MRC) scale, was as follows: ankle dorsiflexion 2/5, plantar flexion 3/5, knee extension 4/5, hip flexion 5/5, intrinsic hand muscles 4−/5, finger extensors 4−/5. Albeit, proximal muscles were predominantly preserved, tongue and neck flexion were unequivocally weak. Deep tendon reflexes were absent throughout.

Spontaneous fasciculations were clinically observed on tongue, facial, upper limb, lower limb muscles and in paravertebral muscles. Mini‐Mental State Examination (MMSE) yielded a score of 26/30, below *z* score −2 according with patient's level of education, consistent with mild cognitive impairment as no daily living activities were impaired.

**FIGURE 1:**
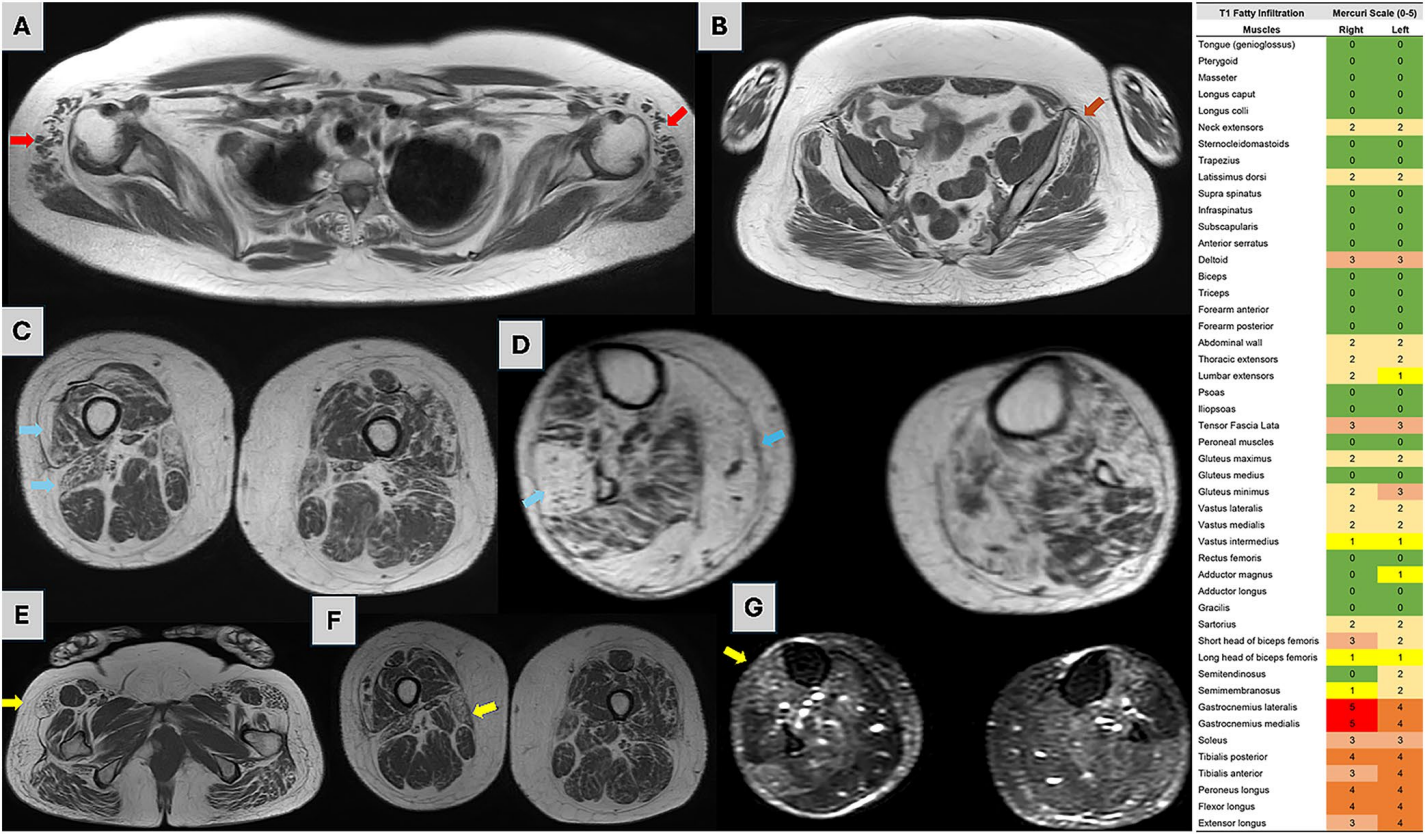
Proband's muscle MRI characterization. Whole‐body muscle MRI (wbMRI) T1 weighted and STIR sequences and fatty infiltration heatmap shows on the upper row, preferential symmetrical deltoid involvement (panel A) and asymmetrical involvement of gluteus minimum (panel B). The pattern depicts the non‐myotomal neither nerve distribution and reveals “muscle islands” suggesting neurogenic origin of abnormalities. In the middle row, preferential involvement of vastus lateralis, biceps femoris short head (panel C), peroneus longus and gastrocnemius medialis (panel D) is shown. Lower row reveals tensor fascia latae involvement with rectus femoris sparing (panel E), sartorius over gracilis involvement (panel F) and STIR hyperintensity on a still viable muscle, tibialis anterior (panel G), and suggesting ongoing denervation. On the right column, a T1wbMRI fatty infiltration heatmap is shown.

Nerve conduction studies performed at age of 53 were consistent with a motor axonal neuropathy, more severe in the lower limbs (Table 1). Needle electromyography revealed increased insertional activity and spontaneous activity at rest, including fibrillation potentials and fasciculations in all muscles evaluated in the cranial, cervical and lumbosacral segments bilaterally. During voluntary contraction muscle action potentials (MUAPs) were reduced in number and had high amplitude, long duration and stable morphology, characterizing chronic denervation and reinnervation in all examined muscles (Table 2; Figure 1). Laboratory investigations revealed elevated serum creatine kinase at 1000 U/L (reference range: 26–192 U/L). Brain MRI was normal.

**TABLE 1:** Nerve conduction studies.

Compound muscle action potential									
Right median SE on APB			Right ulnar SE on ADM			Right and left common peroneal SE on EDB	Right and left posterior tibial SE on AH		
dML, ms (≤ 4.2)	Amp, mV (≥ 3.8)	CV, m/s (≥ 50)	dML, ms (≤ 4.2)	Amp, mV (≥ 4.5)	CV, m/s (≥ 50)	Amp, mV (≥ 2.5)	dML, ms (≤ 5.9)	Amp, mV (≥ 4.5)	CV, m/s (≥ 40)
Median (R)	3.7	0.4	2.5	2.1	52.8	Abs/Abs	4.5/4.9	0.4/0.4	30.6/29.4

Sensory nerve action potentials									
Median R (FII‐W)		Ulnar R (FV‐W)		Radial R (forearm‐W)		Sural R/L (calf‐ankle)		Superficial peroneal R/L (leg‐ankle)	
Amp, μV (≥ 10)	CV, m/s (≥ 50)	Amp, μV (≥ 10)	CV, m/s (≥ 50)	Amp, μV (≥ 15)	CV, m/s (≥ 50)	Amp, μV (≥ 6)	CV, m/s (≥ 40)	Amp, μV (≥ 6)	CV, m/s (≥ 40)
12.3	59.7	13.0	65.2	41.3	61.0	14.4/15.6	41.7/40.2	23.8/22.4	43.6/40.3

Abbreviations: Abs, absent; ADM, abductor digiti minimi; AH, abductor hallucis; Amp, amplitude; APB, abductor pollicis brevis; CV, conduction velocity; DML, distal motor latency; EDB, extensor digitorum brevis; FIll, finger III; FV, finger V; ms, millisecond; SE, surface electrode; W, wrist.

**TABLE 2:** Needle EMG findings.

Muscle	Spontaneous activity	MUAPs	Recruitment	Interference
Paraspinal (lumbar)	DRR, Fasc	Neurogenic	—	—
Orbicularis oris	Fasc	Neurogenic	Reduced	Severely reduced
Mentalis	DRR, Fasc	Neurogenic	Reduced	Severely reduced
Tongue	DRR, Fasc	Neurogenic	Reduced	Severely reduced
Sternocleidomastoid	Fasc	Neurogenic	Reduced	Single unit interference
Deltoid	Fasc	Neurogenic	Reduced	Single unit interference
Extn digitorum com.	Fasc	Neurogenic	Reduced	Severely reduced
Extn Indicis pro.	Fasc	Neurogenic	Reduced	Single unit interference
Pronador Teres	Fasc	Neurogenic	Reduced	Severely reduced
ID	Fibs, PSW, Fasc	Neurogenic	Reduced	Single unit interference
Biceps brachii	Fasc	Neurogenic	Reduced	Severely reduced
Triceps	Fibs, DRR, Fasc	Neurogenic	Reduced	Single unit interference
Vastus medialis	Fasc	Neurogenic	Reduced	Single unit interference
Tibialis anterior	Fasc	Neurogenic	Reduced	Single unit interference
Gastrocnemius (medial)	DRR, Fasc	Neurogenic	Reduced	Severely reduced

Whole‐body muscle MRI (wbMRI) performed at 53 years using T1‐weighted and short tau inversion recovery sequences showed a selective pattern of fatty infiltration. In upper limbs, deltoids were preferentially involved revealing muscle islands, a surrogate of neurogenic involvement [11]. In lower limbs, *gluteus minimu*m was preferentially involved compared to *gluteus medium* and *maximu*m. In the thighs the vastus lateralis, biceps short head, sartorius and tensor fascia latae muscles were preferentially involved, whereas peroneus longus and medial gastrocnemius were more involved in lower legs. STIR hyperintensity was noted in the tibialis anterior, indicating ongoing denervation. This selective pattern, particularly involving gluteus minimum and short head of biceps femoris, suggests a non–length‐dependent neurogenic distribution rather than primary myopathic degeneration.

Whole‐exome sequencing (WES) identified two likely pathogenic heterozygous variants in MCM3AP (NM_003906.5). The first, c.2790‐2A>G (p.?), affects the canonical splice acceptor site of intron 23, replacing the conserved adenine within the invariant AG dinucleotide. Splice‐site prediction algorithms (SpliceAI and MaxEntScan) strongly indicate loss of the natural acceptor site, predicting exon skipping or use of a cryptic splice‐site. Either outcome would likely result in a frameshift and premature termination codon, leading to nonsense‐mediated decay and loss of function. It is absent from gnomAD (0/1445370 alleles). The second variant, c.5884delG, is a single‐base deletion predicted to cause a frameshift (p.Glu1962Lysfs*14) located in exon 25, within the C‐terminal region of the protein and outside the Sac3 domain. This alteration introduces a premature termination codon in the final third of the gene and is expected to trigger nonsense‐mediated mRNA decay, leading to loss of functional protein. This variant is extremely rare (gnomAD: 1/1461876 alleles; frequency 0.000479%), with a PhyloP100 score of 3.642. Both variants are absent from ClinVar and public variant databases and are classified as likely pathogenic according to ACMG criteria and Varsome tool (Richards et al. 2015, [12]). Parental DNA was unavailable for segregation analysis; however, the presence of two pathogenic variants in a gene with established autosomal recessive inheritance, in combination with unaffected non‐consanguineous parents, strongly supports a compound heterozygous configuration.

## 3 Discussion

We describe a patient with complex non‐5q SMA caused by biallelic pathogenic variants in *MCM3AP*, presenting with early‐onset motor weakness, mild cognitive impairment, and electrophysiological evidence of diffuse acute and chronic motor neuronopathy. Whole‐body MRI revealed a selective non‐length dependent but symmetrical pattern of fatty infiltration not previously associated to MCM3AP‐related disorders or to the hereditary lower motor neuronopathies more broadly.

The MCM3AP gene encodes MCM3 acetylase, a GNAT family enzyme involved in DNA replication [13], while also being embedded within the GANP locus, a multifunctional protein implicated in mRNA export via the TREX‐2 complex [14, 15]. This dual role may explain the convergence between neurodegenerative and axonal damage, as impaired mRNA trafficking in motor neurons is a recurrent mechanism across motor neuron diseases. Previous reports have demonstrated that GANP depletion can induce neurodegeneration and even modulate TDP‐43–mediated motor neuron toxicity [16]. Genotype–phenotype correlations suggest that missense variants in the Sac3 domain produce milder neuropathic phenotypes, whereas truncating or splicing variants outside Sac3 result in severe multisystem disease with cognitive involvement and seizures [1, 3].

Our patient carried compound heterozygous variants including a splice‐site mutation in the Sac3 domain (c.2790‐2A>G), predicted to undergo nonsense‐mediated decay, and a frameshift variant in the acetyltransferase domain (p.Glu1962Lysfs14*), which may retain partial protein function. This allelic combination may underlie the relatively attenuated phenotype, with slow progression, absence of sensory involvement, and mild cognitive impairment. This contrasts with previously reported MCM3AP cases, where biallelic loss‐of‐function mutations outside the Sac3 domain produced early, multisystem neurological disease.

Crucially, the neurophysiological findings in this case confound the conventional nosological boundaries between motor neuronopathies. Needle EMG revealed widespread fasciculations, fibrillation potentials and positive sharp waves, indicating active denervation. Such findings are not typically expected in a non‐5q SMA, where the EMG profile usually reflects chronic neurogenic remodeling rather than abundant ongoing denervation [4, 6]. This discrepancy emphasizes the limitations of rigidly classifying inherited motor syndromes. Indeed, emerging evidence suggests that axonopathies and neuronopathies, may exist on a spectrum, with shared molecular pathways contributing to overlapping phenotypes (Kiernan et al. 2021). The coexistence of spontaneous activity with chronic neurogenic remodeling supports a mixed acute‐on‐chronic denervation process, expected in lower motor neuron involvement of ALS rather than pure distal axonopathy.

An additional novel aspect of this case was the muscle wb‐MRI pattern, which revealed disproportionate involvement of the short head of the biceps femoris, sartorius over gracilis, and gluteus minimum over gluteus medium/maximum. Selective patterns of fatty replacement are increasingly recognized as disease‐specific biomarkers in neuromuscular disorders—for example, the characteristic short head of biceps femoris and sartorius involvement in VCP‐related multisystem proteinopathy [17]. This finding suggests that systematic use of MRI may help refine genotype–phenotype correlations in MCM3AP disease and could serve as a diagnostic tool in distinguishing different causes of progressive inherited muscle weakness conditions.

In summary, three major observations emerge from this case. First, it broadens the MCM3AP phenotype to include a slowly progressive, non‐syndromic motor neuronopathy with prominent EMG evidence of active denervation. Second, it illustrates the blurred nosological boundaries between dHMN, non‐5q SMA, and motor neuron diseases, showing that needle EMG findings may contradict clinical‐pathophysiological expectations. Third, the selective muscle MRI pattern observed in this patient may suggest or may even represent a disease‐specific signature for MCM3AP. The evaluation of a greater number of MCM3AP patients is highly expected.

## Data Availability

The data that support the findings of this study are available on request from the corresponding author. The data are not publicly available due to privacy or ethical restrictions.
